# Utilization of Parenteral Morphine by Application of ATC/DDD Methodology: Retrospective Study in the Referral Teaching Hospital

**DOI:** 10.3389/fpubh.2017.00232

**Published:** 2017-08-31

**Authors:** Viktorija Dragojevic-Simic, Nemanja Rancic, Dusica Stamenkovic, Radoje Simic

**Affiliations:** ^1^Centre for Clinical Pharmacology, Medical Faculty Military Medical Academy, University of Defence, Belgrade, Serbia; ^2^Clinic for Anesthesiology and Critical Care, Medical Faculty Military Medical Academy, University of Defence, Belgrade, Serbia; ^3^Department for Plastic Surgery, Institute for Mother and Child Health Care of Serbia “Dr Vukan Cupic”, Medical School, University of Belgrade, Belgrade, Serbia

**Keywords:** drug utilization, opioid analgesics, parenteral morphine, Serbia, surgical patients

## Abstract

**Background:**

Few studies analyzed the pattern of opioid analgesic utilization in hospital settings. The aim of this study was to determine the consumption pattern of parenteral morphine in patients hospitalized in the Serbian referral teaching hospital and to correlate it with utilization at the national and international level.

**Methods:**

In retrospective study, the required data were extracted from medical records of surgical patients who received parenteral morphine in the 5-year period, from 2011 to 2015. We used the Anatomical Therapeutic Chemical Classification/Defined Daily Doses (DDD) international system for consumption evaluation.

**Results:**

While the number of performed surgical procedures in our hospital steadily increased from 2011 to 2015, the number of inpatient bed-days decreased from 2012. However, the consumption of parenteral morphine varied and was not more than 0.867 DDD/100 bed-days in the observed period.

**Conclusion:**

Based on the available data, parenteral morphine consumption in our hospital was lower compared with international data. The low level of morphine use in the hospital was in accordance with national data, and compared with other countries, morphine consumption applied for medical indications in Serbia was low. Adequate legal provision to ensure the availability of opioids, better education and training of medical personnel, as well as multidisciplinary approach should enable more rational and individual pain management in the future, not only within the hospitals.

## Introduction

Surgery causes injury to the body, resulting in an acute pain, and its magnitude is generally correlating to the procedure (1). Acute pain usually resolves when the injury heals (hours to days) (2). However, unrelieved acute postoperative pain has detrimental effects. Adequate pain assessment and management are essential components of perioperative care.

Phenomenon of acute pain is complex and multifactorial. Therefore, analgesia in perioperative settings is optimally achieved by using a multimodal approach with a combination of two or more analgesic medications or modalities with different mechanisms of action, to provide additive or supraadditive synergistic action with fewer adverse effects when compared with a single analgesic medication or modality (3, 4). The concept of the World Health Organization (WHO), analgesic ladder, implies three steps. For mild pain (step 1), a non-opioid analgesic and optional adjuvant are recommended, while in case of moderate pain (step 2), combination of weak opioid with a non-opioid analgesic and optional adjuvant should be used. For severe pain (step 3), combination of strong opioid with a non-opioid analgesic and optional adjuvant are suggested. As far as surgical patients are concerned, it means that the agent or combination of agents should be effective to reduce the pain to an acceptable level, with minimal number of adverse drug reactions, adjusted to the type of surgery, underlying diseases, and cost of therapy. For patients experiencing mild to moderate postoperative pain, local anesthetic wound infiltration, peripheral nerve blockade or administration of non-opioid analgesic, such as an NSAID or acetaminophen, is an appropriate approach (5, 6). For moderate to severe pain after more-invasive surgery, an intravenous (iv) opioid (e.g., morphine, hydromorphone) or epidural containing local anesthetic and opioid is necessary.

Morphine is an archetypal opioid analgetic. Because it is a short-acting opioid, its use has been limited to the management of the acute pain. The frequency of administration necessary to maintain adequate blood level made it difficult to use morphine for chronic pain (7–9). On the other hand, for moderate to severe pain after more-invasive surgery, parenteral administration of morphine is necessary, such as subcutaneous, intramuscular, slow iv injection, or by iv infusion, for example, patient-controlled analgesia (10, 11).

An increasing trend in consumption of opioids has been observed worldwide. However, there are large differences between countries, depending on various factors influencing it (12, 13). In spite of their importance, small number of studies have been performed concerning the pattern of opioid analgesic utilization in hospital settings. The aim of this study was to determine the consumption pattern of parenteral morphine in patients hospitalized in the Serbian referral teaching hospital, during a 5-year period by using Anatomical Therapeutic Chemical Classification/Defined Daily Doses (ATC/DDD) international system and to correlate it with utilization at the national and international level. We hypothesized that the trend in parenteral morphine consumption in our hospital will be positive during 5-year period.

## Materials and Methods

This study was a retrospective, descriptive, and analytical cross-sectional study performed in the Serbian military referral teaching hospital, Military Medical Academy, Belgrade, Serbia, during a 5-year period (from 2011 to 2015).

The data on parenteral morphine consumption in surgical patients of the hospital (intensive care unit) were analyzed according to the ATC/DDD international system for classification and consumption of drugs according to the WHO’s measurement methodology (14). In the ATC, drugs are classified into different groups based on their mechanism of action on organ or organ system and according to the therapeutic, pharmacological, and chemical properties. DDD values are actually the assumed average maintenance dose per day for a drug used for its main indication for an average adult. DDD per 100 bed-days was used as a measurement unit for analyzing consumption of parenterally administered morphine. It was calculated by using the following equation:

$$\text{DDD/100> bed/day}=\frac{\text{Consumed quantity per year (mg)/DDD}}{365\times \text{Number of beds }\times \text{Occupancy index}}\times 100.$$

The amount for parenteral morphine of standard DDD and ATC code is 30 mg and N02AA01. In the surgical intensive care, morphine was used per protocol, as continuous iv infusion at the dose of 0.01–0.05 mg/kg/h, with iv bolus 1 mg if numeric rating scale is higher than 4. The indications for systemic morphine postoperative analgesia were major abdominal, vascular, and thoracic surgery, polytrauma cases in case of epidural catheter absence, or malfunction.

Microsoft Office Excel 2007 software was used for statistical analysis. Data from our hospital during the period from 2011 to 2015 were used for trend analysis. The continual variables were presented as mean value ± SD.

## Results

During the observed 5-year period, 21,971 patients were operated on and treated by parenteral formulation of morphine. The mean (±SD) number of treated patients per year was 4,394.20 (±385.79).

Table 1 shows distribution of all performed surgical procedures per year and the number of bed-days for the whole hospital, as well as the calculated number of bed-days per surgical procedures. The number of surgical procedures was steadily increasing during the whole observed period, from 2011 to 2015. However, the number of inpatient bed-days and the number of bed-days per surgical procedures decreased from 2013 onward.

**Table 1:** Number of surgical procedures and patient bed-days during the examined 5-year period in the referral, teaching hospital.

Year	Number of surgical procedures	Inpatient bed-days	BD/s. proc.
2011	13,750	301,154	21.902
2012	14,555	310,433	21.328
2013	14,736	307,467	20.865
2014	15,752	286,885	18.213
2015	15,673	263,600	16.819
M ± SD	14,893.2 ± 835.4	293,907.8 ± 19217.9	19.825 ± 2.169

*BD/s. proc., bed-days per surgical procedures*.

**Figure 1:**
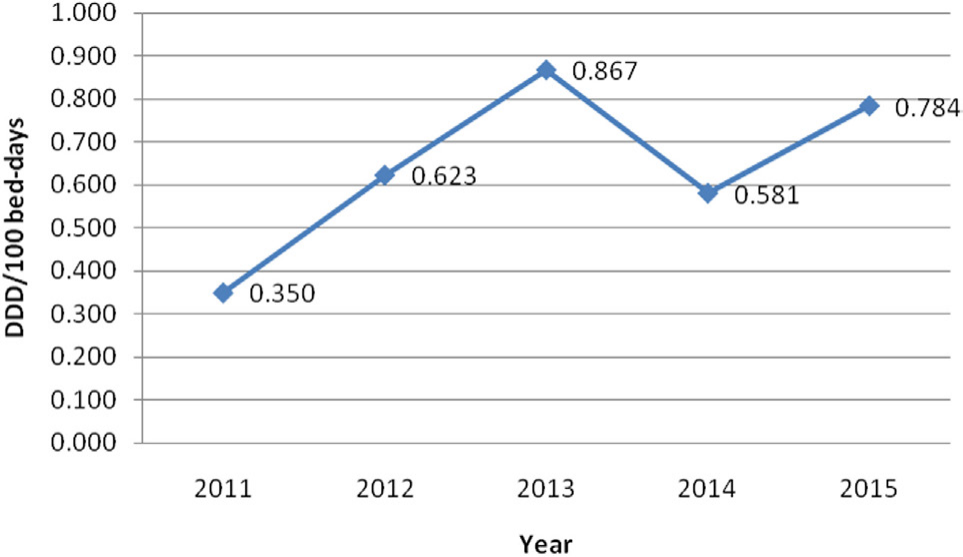
The trend of parenteral morphine consumption based on defined daily doses (DDD)/100 bed-days unit during the examined 5-year period.

**Figure 2:**
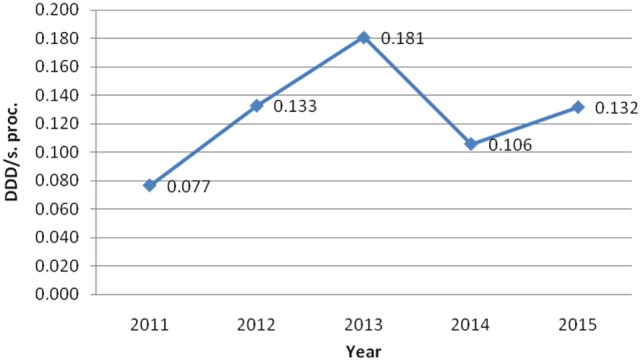
The trend of parenteral morphine consumption based on its number of defined daily doses per surgical procedure (DDD/s. proc.) during the examined 5-year period.

On the other hand, Table 2 shows consumed amount of parenteral morphine based on DDD and DDD/100 bed-days units during examined 5-year period. The 5-year trend of parenteral morphine consumption is also demonstrated in Figures 1 and 2. The average number of DDD for morphine consumption as well as the average (mean ± SD) number of DDD/100 bed-days and DDD per surgical procedures increased from 2011 to 2013, then dropped in 2014, to 0.581 DDD/100 bed-days, and increased again to the value of 0.784 DDD/100 bed-days.

**Table 2:** The consumed amount of parenteral morphine in the hospital during the 5-year study period.

Year	Morphine consumption (mg)	Morphine consumption (DDD)	DDD/100 bed-days	DDD/s. proc.
2011	31,600	1,053.33	0.350	0.077
2012	58,000	1,933.33	0.623	0.133
2013	80,000	2,666.67	0.867	0.181
2014	50,000	1,666.67	0.581	0.106
2015	62,000	2,066.67	0.784	0.132
M ± SD	56,320.0 ± 17,655.4	1,877.3 ± 588.5	0.641 ± 0.200	0.125 ± 0.038

*DDD, defined daily doses; s. proc., surgical procedures*.

Taking into account all obtained data, it can be seen that although the number of performed surgical procedures in our hospital steadily increased from 2011 to 2015, the number of inpatient bed-days decreased from 2012, onward. However, the consumption of parenteral morphine varied and did not completely follow this trend, although it was positive.

## Discussion

We partly confirmed working hypothesis of our study, since there was the positive trend in the consumption of parenteral morphine in our hospital, although not during the whole observed 5-year period. Drug utilization studies are important as they provide valuable data analyses for particular time period, heath care setting or population, as a whole (15–18). Moreover, we can evaluate the effects attained by therapeutic interventions (12). The various patterns of drug use should enable making national or international comparison, and, if necessary, policy changes (13). An increasing trend in the consumption of opioids has been observed worldwide in the last two decades (19, 20). However, according to the WHO data from year 2003, six developed countries accounted for 79% of global morphine consumption, while developing countries only accounted for 6% (19, 20). North American and Western European countries consume the majority of the morphine produced in the world even though the combined population of these areas is 17.2% of the world’s population (19, 20). However, European countries themselves had different trends in the opioid utilization. Study performed from 2002 to 2006 showed increased consumption of opioids in all Scandinavian countries, except Sweden, concerning oxycodone, fentanyl, and methadone (21, 22). Morphine had decreasing consumption trend (21, 22).

Opioid consumption level in Spain, Italy, Greece, and Croatia was significantly lower than in Germany, Austria, Belgium, and Denmark (22–24). Our previous investigation showed that the total opioid analgesic consumption in Serbia, in 2012 and 2013, was 8.83 and 8.55 times lower than in Croatia, respectively (25). Despite high opioid consumption in world’s six high developed countries, data show that only 24% patients with pain in these countries were adequately managed (26). Despite an increased focus on pain management programs, many patients continue to experience intense postoperative pain (27, 28). Studies carried out in Danish hospitals revealed that the intensity of pain was not evaluated in 55% of patients on postoperative day 1, in 71% on postoperative day 2, and in 84% on postoperative day 3 (29, 30). Findings indicated uncontrolled pain in 45.5% of patients. Patients who experienced severe pain received only 50% of available strong opioids and 73.3% of available weak opioids (29, 30). Despite the available guidelines for acute pain management, which are evidence based and available for free download, only in 14% of patients, pain is treated based on guidelines (27). Similar reports can be found from the US hospitals (31).

Parenteral morphine consumption in our hospital was much lower in the observed period from 2011 to 2015 and not more than 0.867 DDD/100 bed-days. It is difficult to comment our data, as only few drug utilization evaluation studies concerning the consumption of parenteral opioids in hospital settings, based on the ATC/DDD system, were published (32, 33). Study from Spain evaluated the use of parenteral opioid analgesics in one university general hospital (32). During 4-year period (2004–2008), consumed amount of morphine was 3.59, 3.73, 3.6, and 4.3 DDD/100 bed-days, respectively (32). According to data from referral teaching hospital in Iran, parenteral morphine consumption was 7.83 DDD/100 bed-days during year 2013 (33). On the basis of these data, we had significantly lower parenteral consumption then published studies. One reason was the fact that parenteral morphine was prescribed only for pain management in patients hospitalized at the surgical intensive care unit. In the previously mentioned studies, parenteral morphine was prescribed for patients at surgical wards and internal medicine wards. It is impossible to assess the quality of pain management at the ward for our hospital, as we do not have patient reported data. What is known that only non-steroid anti-inflammatory medications per patient request were used at the ward for postoperative pain management, even on the first day after major surgeries including abdominal, vascular, and cardiothoracic surgeries. Based on our experience, it is obvious that the lack of knowledge and interest to implement particular medications among medical staff is the root of the problem. Above all, the non-existence of acute pain team makes pain management and assessment on the ward more difficult.

The low level of morphine use in our hospital was in accordance with national data, which show that the consumed amounts of parenteral morphine in the period from 2012 to 2015 in Serbia were 0.015, 0.005, 0.010, and 0.009 DDD per 1,000 inhabitants per day, respectively, according to the Medicines and Medical Devices Agency of Serbia (34). As it was already mentioned, total opioid analgesic consumption in Serbia, in 2012 and 2013, was even 8.83 and 8.55 times lower than in Croatia, respectively (25). In general, consumption of morphine in Croatia was higher in comparison to Serbia, according to data in 2007 onward (22, 34–36). Moreover, study performed in nine western European countries (Belgium, Germany, Italy, Norway, Spain, Ireland, United Kingdom, Portugal, and Netherlands) has shown that the consumption of morphine was the lowest in all countries in comparison to other opioid analgesics (fentanyl, tramadol, and codeine); morphine consumption was about three times lower comparing with the consumption of transdermal fentanyl (37). Even in some hospitals in the United States, parenteral morphine boluses are routinely given at lower doses compared with other opioids or patient-controlled analgesic dosing (38). This habit can be a reason for inadequate patient analgesia (38). Unfortunately, major factors for inadequate opioid dosage and treatment duration are unjustified fears of adverse opioid effects, including abuse, insufficient knowledge of opioid characteristics and insufficient training of medical staff.

The authors who have dealt with opioid purchasing and costs even relates low consumption of parenteral morphine with the fact that international non-proprietary name morphine, unlike other opioids, has not known brand name linkable to the this chemical compound or is of less interest in marketing due to its low market price (37). Serbia has experienced profound transformation concerning value-based medication consumption and expenditure during socioeconomic transition, due to an extreme combination of unfavorable events in the recent past (39). Consequently, difficulties in provision of sustainable financing and increasingly frequent shortages of pharmaceuticals are present (40). Above all, medication tenders have been shown as resource consuming, laborious, and risky job, which need time for adequate conducting (41). All of these facts influenced the purchasing and consumption of parenteral morphine at the Serbian market as a whole, as well as in our referral teaching hospital.

The limitation of our study is the fact that we have not evaluated the trend in the consumption of other opioid drugs in the hospital and compared it with parenteral morphine. However, there are not many studies in worldwide dealing with this issue, based on ATC/DDD international system. Therefore, we started with consideration of parenteral morphine, one of the rarest used analgesics in our hospital, to evaluate its consumption. On the basis of our experience, we can achieve better pain management program by organized multidisciplinary team consisting of anesthesiologists, neurologists, neurosurgeons, and clinical pharmacologists, with educated medical staff.

## Conclusion

Parenteral morphine consumption in our hospital was much lower in comparison with available data concerning other hospitals, as result of limited opioid prescription. Although the number of surgical procedures was steadily growing, the parenteral morphine consumption did not follow this trend all the time. The low level of morphine use in our hospital was in accordance with national data. Comparison with other countries indicated that morphine consumption applied for medical indications in Serbia was low. Adequate provision to ensure and not to unduly restrict the availability of drugs that are considered indispensable for medical and scientific purposes, such as opioids, is necessary within each country. Better education and training of medical staff, as well as multidisciplinary approach, should enable rational medication application and patient-tailored pain management in the future, not only within the hospitals.

## Author Contributions

All the authors listed have made substantial contribution to the conception, design, analysis, and interpretation of data for the work and approved it for publication. NR, DS, and VD-S developed research questions, designed the study, and prepared the manuscript. NR, DS, RS, and VD-S searched the literature and analyzed and interpreted the data for the work. VD-S and DS critically reviewed the manuscript.

## Conflict of Interest Statement

The authors declare that the research was conducted in the absence of any commercial or financial relationships that could be construed as a potential conflict of interest.

## 

Funding

## References

[1] Turk DC Okifuji A Pain terms and taxonomies of pain. In: Loeser JD Butler SH Chapman CR Turk DC, editors. Bonica’s Management of Pain. Philadelphia: Lippinkott Williams &Wilkins (2000). p. 17–25.

[2] Donelly AJ Golembiewski JA Rakic AM Perioperative care. In: Alldredge BK Corelli RL Ernst ME Guglielmo BJ Jacobson PA Kradjan WA Williams BR, editors. Koda-Kimble Young’s Applied Therapeutics: The Clinical Use of Drugs. Philadelphia: Lippinkott Williams &Wilkins, A Wolters Kluwer Business (2013). p. 147–74.

[3] Chou R Gordon DB de Leon-Casasola OA Rosenberg JM Bickler S Brennan T Management of postoperative pain: a Clinical Practice Guideline from the American Pain Society, the American Society of Regional Anesthesia and Pain Medicine, and the American Society of Anesthesiologists’ Committee on Regional Anesthesia, Executive Committee, and Administrative Council. J Pain (2016) 17(2):131–57. 10.1016/j.jpain.2015.12.008 26827847

[4] Grond S Sablotzki A. Clinical pharmacology of tramadol. Clin Pharmacokinet (2004) 43(13):879–923. 10.2165/00003088-200443130-00004 15509185

[5] O’Neil CK Pain management. 2nd ed In: Chisholm-Burns MA Schwinghammer TL Wells BG Malone PM Kolesar JM DiPiro JT, editors. Pharmacotherapy: Principles & Practice. New York: McGraw-Hill Companies, Inc. (2010). p. 567–81.

[6] Kral LA Ghafoor VL Pain and its management. 10th ed In: Alldredge BK Corelli RL Ernst ME Guglielmo BJ Jacobson PA Kradjan WA Williams BR, editors. Koda-Kimble & Young’s Applied therapeutics: The Clinical Use of Drugs. Philadelphia: Lippincott Williams & Wilkins, A Wolters Kluwer Business (2013). p. 112–46.

[7] Balch RJ Trescot A. Extended-release morphine sulfate in treatment of severe acute and chronic pain. J Pain Res (2010) 3:191–200. 10.2147/JPR.S6529 21197323 PMC3004644

[8] Yaksh TL Wallace MS Opioids, analgesia, and pain management. 12th ed In: Brunton LL, editor. Goodman and Gilman’s The Pharmacological Basis of Therapeutics. New York: McGraw-Hill Companies, Inc. (2010). p. 481–525.

[9] Argoff CE Silvershein DI. A comparison of long- and short-acting opioids for the treatment of chronic noncancer pain: tailoring therapy to meet patient needs. Mayo Clin Proc (2009) 84(7):602–12. 10.1016/S0025-6196(11)60749-0 19567714 PMC2704132

[10] McEvoy GK, editor. AHFS Drug information 2011. Bethesda: American Society of Health-System Pharmacist (2011). 3823 p.

[11] BMJ Group. British National Formulary 70. London: BMJ Group and RPS Publishing (2009). 1366 p.

[12] Natsch S Hekster YA de Jong R Heerdink ER Herings RM van der Meer JW. Application of the ATC/DDD methodology to monitor antibiotic drug use. Eur J Clin Microbiol Infect Dis (1998) 17(1):20–4. 10.1007/BF01584358 9512177

[13] International Narcotics Control Board (INCB): Narcotic Drugs Technical Report: Estimated World Requirements for 2013. New York: United Nations (2013). Available from: https://www.incb.org/documents/Narcotic-Drugs/Technical-Publications/2012/Narcotic_Drugs_Report_2012.pdf

[14] WHO Collaborating Centre for Drug Statistics Methodology: Definition and General Consideration. (2013). Available from: https://www.whocc.no/filearchive/publications/1_2013guidelines.pdf

[15] Dukes MNG, editor. Drug Utilization Studies: Methods and Uses. Finland: WHO Regional Publications (1993). 221 p. European Series No. 45. 8442841

[16] Alsirafy SA Ibrahim NY Abou-Elela EN Opioid consumption before and after the establishment of a palliative medicine unit in an Egyptian cancer centre. J Palliat Care (2012) 28(3):135–40. 23098011

[17] Zin CS Chen LC Knaggs RD. Changes in trends and pattern of strong opioid prescribing in primary care. Eur J Pain (2014) 18(9):1343–51. 10.1002/j.1532-2149.2014.496.x 24756859 PMC4238849

[18] Berterame S Erthal J Thomas J Fellner S Vosse B Clare P Use of and barriers to access to opioid analgesics: a worldwide, regional, and national study. Lancet (2016) 387(10028):1644–56. 10.1016/S0140-6736(16)00161-6 26852264

[19] Duthley B Asholten W Adequacy of opioid analgesic consumption at country, global, and regional level in 2010, its relationship with development level, and changes compared with 2006. J Pain Symptom Manage (2014) 47(2):283–97. 10.1016/j.jpainsymman.2013.03.015 23870413

[20] Manjiani D Paul DB Kunnumpurath S Kaye AD Vadivelu N. Availability and utilization of opioids for pain management: global issues. Ochsner J (2014) 14(2):208–15. 24940131 PMC4052588

[21] Hamunen K Laitinen-Parkkonen P Paakkari P Breivik H Gordh T Jensen NH What do different databases tell about the use of opioids in seven European countries in 2002? Eur J Pain (2008) 12(6):705–15. 10.1016/j.ejpain.2007.10.012 18162422

[22] Krnic D Anic-Matic A Dosenovic S Draganic P Zezelic S Puljak L National consumption of opioid and nonopioid analgesics in Croatia: 2007–2013. Ther Clin Risk Manag (2015) 11:1305–14. 10.2147/TCRM.S86226 26357478 PMC4559243

[23] García del Pozo J Carvajal A Rueda de Castro AM Cano del Pozo MI Martín Arias LH Opioid consumption in Spain – the significance of a regulatory measure. Eur J Clin Pharmacol (1999) 55(9):681–3. 10.1007/s002280050693 10638399

[24] International Narcotics Control Board. Availability of Internationally Controlled Drugs: Ensuring Adequate Access for Medical and Scientific Purposes Indispensable, Adequately Available and Not Unduly Restricted. New York, NY: United Nations (2016). Available from: http://www.unis.unvienna.org/unis/protected/2016/INCB_availability_report.pdf

[25] Rančić N Stamenković D Dragojević-Simić V Opioid analgesic consumption in Serbia during two years period (opioid analgesic consumption in Serbia) (In Serbian, English abstract). SJAIT (2016) 38(5–6):145–53. 10.5937/sjait1606145R

[26] International Narcotics Control Board for 2007. New York, NY: United Nations (2008). Available from: https://www.incb.org/documents/Publications/AnnualReports/AR2007/AR_07_English.pdf

[27] Meissner W Coluzzi F Fletcher D Huygen F Morlion B Neugebauer E Improving the management of post-operative acute pain: priorities for change. Curr Med Res Opin (2015) 31(11):2131–43. 10.1185/03007995.2015.1092122 26359332

[28] Sommer M de Rijke JM van Kleef M Kessels AG Peters ML Geurts JW The prevalence of postoperative pain in a sample of 1490 surgical inpatients. Eur J Anaesthesiol (2008) 25(4):267–74. 10.1017/S0265021507003031 18053314

[29] Lorentzen V Hermansen IL Botti M. A prospective analysis of pain experience, beliefs and attitudes, and pain management of a cohort of Danish surgical patients. Eur J Pain (2012) 16(2):278–88. 10.1016/j.ejpain.2011.06.004 22323380

[30] Misiołek H Cettler M Woroń J Wordliczek J Dobrogowski J Mayzner-Zawadzka E The 2014 guidelines for post-operative pain management. Anaesthesiol Intensive Ther (2014) 46(4):221–44. 10.5603/AIT.2014.0041 25293474

[31] Apfelbaum JL Chen C Mehta SS Gan TJ. Postoperative pain experience: results from a national survey suggest postoperative pain continues to be undermanaged. Anesth Analg (2003) 97(2):534–40. 10.1213/01.ANE.0000068822.10113.9E 12873949

[32] Gómez Salcedo P Herrero Ambrosio A Muñoz y Ramón JM Estudio de utilización de analgésicos opiáceos en un hospital general universitario. Rev Soc Esp Dolor (2009) 16(7):373–80. 10.1016/S1134-8046(09)72817-4

[33] Soltani R Vatanpour H Shafiee F Sadeghian N Evaluation of parenteral opioid analgesics utilization in patients hospitalized in a referral teaching hospital. J Pharm Care (2015) 3(1–2):16–20.

[34] Medicines and Medical Devices Agency of Serbia. Publication. Marketing and Consumption of Medicinal Product for Human Use (In Serbian). (2017). Available from: https://www.alims.gov.rs/eng/about-agency/publication

[35] Index mundi. Croatia vs. Serbia. (2017). Available from: http://www.indexmundi.com/factbook/compare/croatia.serbia

[36] Agency for Medicines and Medical Devices HALMED. Consumption of Drugs in Croatia from 2009 to 2013 (In Croatian). (2015). Available from: http://www.almp.hr/fdsak3jnFsk1Kfa/publikacije/Potrosnja-lijekova-u-RH_2009-2013

[37] De Conno F Ripamonti C Brunelli C. Opioid purchases and expenditure in nine western European countries: ‘are we killing off morphine?’ Palliat Med (2005) 19(3):179–84. 10.1191/0269216305pm1002oa 15920930

[38] O’Connor AB Lang VJ Quill TE. Underdosing of morphine in comparison with other parenteral opioids in an acute hospital: a quality of care challenge. Pain Med (2006) 7(4):299–307. 10.1111/j.1526-4637.2006.00183.x 16898939

[39] Garfield R Economic sanctions on Yugoslavia. Lancet (2001) 358(9281):580 10.1016/S0140-6736(01)05713-0 11520550

[40] Jakovljevic MB Djordjevic N Jurisevic M Jankovic S. Evolution of the Serbian pharmaceutical market alongside socioeconomic transition. Expert Rev Pharmacoecon Outcomes Res (2015) 15(3):521–30. 10.1586/14737167.2015.1003044 25592856

[41] Milovanovic DR Pavlovic R Folic M Jankovic SM. Public drug procurement: the lessons from a drug tender in a teaching hospital of a transition country. Eur J Clin Pharmacol (2004) 60(3):149–53. 10.1007/s00228-004-0736-1 15057496

